# Identification of Persistent Sulfidogenic Bacteria in Shale Gas Produced Waters

**DOI:** 10.3389/fmicb.2020.00286

**Published:** 2020-02-21

**Authors:** Lisa Cliffe, Sophie L. Nixon, Rebecca A. Daly, Bob Eden, Kevin G. Taylor, Christopher Boothman, Michael J. Wilkins, Kelly C. Wrighton, Jonathan R. Lloyd

**Affiliations:** ^1^Department of Earth and Environmental Sciences, The University of Manchester, Manchester, United Kingdom; ^2^Department of Soil and Crop Sciences, College of Agricultural Sciences, Colorado State University, Fort Collins, CO, United States; ^3^Rawwater Engineering Company Limited, Culcheth, United Kingdom

**Keywords:** thiosulfate-reducing bacteria, biogenic sulfide, *Halanaerobium*, non-conventional gas, microbiology

## Abstract

Produced waters from hydraulically fractured shale formations give insight into the microbial ecology and biogeochemical conditions down-well. This study explores the potential for sulfide production by persistent microorganisms recovered from produced water samples collected from the Marcellus shale formation. Hydrogen sulfide is highly toxic and corrosive, and can lead to the formation of “sour gas” which is costly to refine. Furthermore, microbial colonization of hydraulically fractured shale could result in formation plugging and a reduction in well productivity. It is vital to assess the potential for sulfide production in persistent microbial taxa, especially when considering the trend of reusing produced waters as input fluids, potentially enriching for problematic microorganisms. Using most probable number (MPN) counts and 16S rRNA gene sequencing, multiple viable strains of bacteria were identified from stored produced waters, mostly belonging to the Genus *Halanaerobium*, that were capable of growth via fermentation, and produced sulfide when supplied with thiosulfate. No sulfate-reducing bacteria (SRB) were detected through culturing, despite the detection of relatively low numbers of sulfate-reducing lineages by high-throughput 16S rRNA gene sequencing. These results demonstrate that sulfidogenic produced water populations remain viable for years post production and, if left unchecked, have the potential to lead to natural gas souring during shale gas extraction.

## Introduction

Shale gas is an unconventional hydrocarbon being utilized increasingly as a cleaner alternative to coal (Jenner and Lamadrid, 2013). Natural gas is recovered from shale formations by means of hydraulic fracturing; the high pressure injection of water-based fluids to force open fractures in the rock, allowing gas to flow to the surface (Lee et al., 2011; Mouser et al., 2016). Input fluids can then interact with the shale formation leading to changes in the geochemistry of the produced waters (Schramm, 2011; Murali Mohan et al., 2013a; Akob et al., 2015). Studies on produced waters from the Marcellus shale show an inverse correlation between dissolved organic carbon and salinity (Cluff et al., 2014), with salinity increasing with time (Hayes, 2009; Schramm, 2011; Barbot et al., 2013; Murali Mohan et al., 2013a; Cluff et al., 2014; Akob et al., 2015; Rowan et al., 2015). Fracturing fluids typically include sand and a number of organic and inorganic additives, to hold open newly formed fractures and allow operations to run as efficiently as possible (Stringfellow et al., 2014; Elsner and Hoelzer, 2016). Included among common additives are biocides, used to prevent microbial activity from interfering with gas extraction (Elsner and Hoelzer, 2016). Despite the addition of biocides and the extreme conditions encountered downhole including high salinity, pressure, reducing conditions, and moderately high temperatures (Fichter et al., 2012; Akob et al., 2015; Daly et al., 2016; Nixon et al., 2019) prior studies have identified microbial communities in produced waters from fractured shales in the Antrim, Barnett, Burkett, Utica, Bakken, and Marcellus formations in the United States (Davis et al., 2012; Struchtemeyer and Elshahed, 2012; Murali Mohan et al., 2013a, b; Strong et al., 2013; Wuchter et al., 2013; Cluff et al., 2014; Akob et al., 2015; Daly et al., 2016; Liang et al., 2016; Lipus et al., 2017; Evans et al., 2018) as well as the Sichuan Basin in China (Zhang et al., 2017). A common finding in many of these studies, is a transition from high diversity microbial communities in injected fluids, resembling the freshwater environment the water was sourced from, to an enriched and lower diversity community of mostly halotolerant and halophilic facultative and strict anaerobes, such as *Halanaerobium* (Davis et al., 2012; Struchtemeyer and Elshahed, 2012; Murali Mohan et al., 2013a; Strong et al., 2013; Cluff et al., 2014; Akob et al., 2015; Daly et al., 2016; Mouser et al., 2016; Vikram et al., 2016; Booker et al., 2017; Lipus et al., 2017; Borton et al., 2018; Evans et al., 2019).

Some halotolerant microorganisms common to fractured shales can impact negatively on shale gas extraction. Microbial colonization of fractured shale may lead to sour gas production, microbially induced corrosion and bioclogging, with implications for both human safety and well productivity. Metabolic by-products such as organic acids and to a greater extent hydrogen sulfide can corrode steel infrastructure leading to environmental hazards and lost revenue (Eden et al., 1993). Hydrogen sulfide is also highly flammable, toxic to human health (Guidotti, 2010) and can result in “sour gas” which is costly to refine (Eden et al., 1993; Fichter et al., 2012). Sulfide may be produced through the reduction of sulfur species (such as sulfate and thiosulfate) coupled to the direct oxidation of organics or hydrogen by sulfate-reducing (Grein et al., 2013) and thiosulfate-reducing bacteria (TRB) (Ravot et al., 1997). Alternatively biogenic sulfide can also result from the transfer of reducing equivalents onto thiosulfate during fermentation reactions (Booker et al., 2017). Additionally, the formation of microbial biofilms may impede gas flow and result in fracture clogging (Bottero et al., 2010). Evidence suggests that organic chemicals used during hydraulic fracturing may stimulate these deleterious processes, for example via organic acid production from fermentation reactions, and sulfide production from sulfate and thiosulfate reduction pathways (Struchtemeyer et al., 2011; Liang et al., 2016; Booker et al., 2017, 2019; Nixon et al., 2017; Evans et al., 2019). Sulfate-reducing bacteria (SRB) have been identified previously in production fluids through next generation sequencing but their *in situ* role in sulfide production is undetermined (Davis et al., 2012; Struchtemeyer and Elshahed, 2012; Liang et al., 2016), whereas TRB have been isolated from these environments and represent an alternative route to sulfide production (Ravot et al., 1997, 2005; Liang et al., 2016; Booker et al., 2017; Lipus et al., 2017).

Microorganisms in production fluids could prove especially problematic when considering the trend for their reuse in future fracturing operations. This practice reduces the demand on freshwater supplied and circumvents challenges related to disposal (Gregory et al., 2011; Murali Mohan et al., 2013b; Cluff et al., 2014). Produced waters are often stored above ground in impoundments or sealed tanks prior to reuse or disposal, undergoing aeration or biocide treatment prior to reuse, with variable success in regard to microbial control (Murali Mohan et al., 2013b). It is therefore essential to understand the potential for stored produced waters to cause further microbial problems in shale gas extraction. However, relatively little is known about the metabolism of viable microorganisms in production waters (Daly et al., 2016; Vikram et al., 2016; Booker et al., 2017). Given the potential for deleterious microbial processes to affect shale gas extraction, there remains a critical need to assess the metabolic capabilities and resilience of persistent taxa recovered from hydraulically fractured shale systems. Here, we assess the persistence of problematic microorganisms in produced waters collected from a shale gas well in the Marcellus formation. Samples were stored for several 100 days between sampling and the analysis reported here, giving a good view of the potential role of persistent microbes present in souring reactions. Culture based approaches were used to investigate the presence of sulfate-reducing, thiosulfate-reducing and fermentative bacteria (FB), and the composition of most probable number (MPN) enrichment cultures for these organisms was characterized and compared to the microbial composition of the produced fluids soon after collection, to identify the most persistent taxa. Collectively, these data confirm the numerical dominance and long-term persistence of fermentative TRB, alongside a far lower number of SRB, highlighting the importance of developing appropriate diagnostic tools to help control microbial sulfide production.

## Materials and Methods

### Sampling

Produced water samples were collected over several 100 days as part of a Marcellus Shale Energy and Environment Laboratory project (MSEEL, 2015) in order to characterize the changes to production fluid geochemistry and microbiology, and full details of sample collection have been published previously (Evans et al., 2018; Daly et al., 2019). Prior research has shown that over this period of time, produced fluids become more saline and the community less diverse, with halotolerant bacteria dominating later stage fluids (Daly et al., 2016). Produced water samples from the gas-water-separator of the MIP-3H shale gas well in West Virginia (herein known as Marcellus-4) were collected daily. The authors had access to stored samples collected at 29, 79, 142, and 303 days after hydraulic fracturing. These samples were collected between December 2015 and September 2016 and selected for use in this study representing a spread from early flowback to late production fluids. Samples from the same well have been reported on in prior studies (Evans et al., 2018; Daly et al., 2019) Prior to use in this study, samples were stored at room temperature under anoxic conditions for varying periods of time. The first set of experiments were conducted on samples stored for 808, 758, 695, and 534 days, respectively. Produced water samples were stored for an additional 291 days prior to the second set of MPN experiments conducted using TRB l medium with the addition of yeast extract.

### Most Probable Number (MPN) Counts

Most probable number counts were used to estimate the number of viable heterotrophic, sulfate-reducing and thiosulfate-reducing microorganisms in the four stored produced water samples, all dilution series were run in triplicate and incubated at 40°C, comparable to previously reported temperatures (Akob et al., 2015) for up to 112 days; the most positive endpoints then underwent 16S rRNA sequencing. Strict anaerobic techniques were used throughout (Miller and Wolin, 1974). A starting dilution of 1 ml inoculum into 9 ml media was diluted ten-fold in serial dilutions to a final concentration of 1:10^7^this was repeated for each sample in triplicate. The salinities of the selective media were chosen to reflect the high salinities previously measured in produced fluid samples (Daly et al., 2016). Growth was identified visually based on increased turbidity (fermentative bacteria MPNs) or the presence of a black precipitate (iron sulfide formation in sulfate-reducing and thiosulfate-reducing MPNs). MPN counts were then calculated from the vials scored as positive (Oblinger and Koburger, 1975). When estimating MPN counts for sulfate-reducing and thiosulfate-reducing cultures a pitch-black vial (compared to the colorless control) was deemed a positive result. Sulfate-reducing and thiosulfate-reducing media were amended with iron (II) chloride, which reacts with sulfide to form a black FeS precipitate in the presence of sulfide, an anticipated by-product of microbial thiosulfate- and sulfate-reduction. Addition of sodium sulfide as a reducing agent would lead to false positive results, and therefore we opted to add sodium thioglycolate to SRB media instead [as in Postgate media (Tanner, 1989)]. The fermentative medium lacked iron(II) chloride and hence sodium sulfide was used as a reducing agent. For the FB a positive score was based on visible turbidity, compared to the control. Since this medium contained resazurin (a redox-sensitive indicator dye), optical density readings could not be conducted aerobically. MPN enrichment media are described below.

### Fermentative Bacteria (FB) Medium

This medium was adapted from Spring et al. (2015) to target viable FB in the produced water samples (Spring et al., 2015). The medium contained (in grams per liter deionized water unless otherwise stated): NaCl (80.0), MgCl_2_.6H_2_O (6.0), NH_4_Cl_2_ (1.0), CaCl.2H_2_O (0.4), K_2_HPO_4_ (0.4), KCl (1.5), yeast extract (0.2), Na_2_CO_3_ (0.75), L-Cysteine- HCL- H_2_O (0.3), D-glucose (2.0), and 10 ml vitamin solution. The vitamin solution contained (in mg per liter deionized water) biotin (2.0), folic acid (2.0), pyridoxine-HCl (10.0), riboflavin (5.0), thiamine (5.0), nicotinic acid (5.0), pantothenic acid (5.0), vitamin B-12 (0.1), p-aminobenzoic acid (5.0), and thioctic acid (5.0). The medium was dispensed into 10 ml glass serum vials, purged with an 80:20 mix of N_2_:CO_2_ for 10 min, and autoclaved at 121°C for 20 min. Prior to inoculation, all FB aliquots were amended with 1.25 mM sodium sulfide from a sterile anoxic stock and the pH adjusted to 7.3–7.5.

### Sulfate-Reducing Bacteria (SRB) Medium

This medium was modified from the Postgate B recipe (Tanner, 1989) and contained (in grams per liter deionized water): KH_2_PO_4_ (0.5), CaSO_4_.2H_2_O (1.26), NH_4_Cl (1.00), MgSO_4_.7H_2_O (2.00), Yeast extract (1.00), FeSO_4_.7H_2_O, ascorbic acid (0.10), sodium thioglycolate (0.10), sodium acetate (2.56), sodium lactate (3.5), and NaCl (100.00). The pH was adjusted to 7.1, before being dispensed, purged with N_2_:CO_2_ and autoclaved as above.

### Thiosulfate-Reducing Bacteria (TRB) Medium

This bespoke medium contained (in grams per liter deionized water): NaHCO_3_ (2.5), NH_4_Cl (0.25), NaH_2_PO_4_H_2_O (0.06), KCl (0.1), sodium acetate (2.56), sodium lactate (3.5) NaCl (100), Na_2_S_2_O_3_.5H_2_O (4.27), 10 ml vitamin solution (as above), and 10 ml mineral solution. The mineral solution contained (in mg per liter deionized water) nitrilotriacetic acid (1.5), MgSO_4_ (3.0), MnSO_4_.H_2_O (0.5), NaCl (1.0), FeSO_4_.7H_2_O (0.1), CaCl_2_.2H_2_O (0.1), CoCl_2_.6H_2_O (0.1), ZnCl_2_ (0.13), CuSO_4_.5H_2_O (0.01), AlK (SO_4_)_2_.12H_2_O (0.01), H_3_BO_3_ (0.01), NaMoO_4_ (0.025), NiCl_2_.6H_2_O (0.024), and NaWO_4_.2H_2_O (0.025). The medium was dispensed, purged with N_2_:CO_2_ and autoclaved as above. Filter-sterilized iron (II) chloride was then added from anoxic stock to a final concentration of 1.8 mM (as an indicator for sulfide production)and the pH adjusted to 6.8–7.0. Subsequent TRB MPNs were repeated with the addition of 0.2 g/L yeast extract to the medium prior to autoclave, which was otherwise prepared as above.

### Ion Chromatography

Acetate, lactate and thiosulfate concentrations in MPN cultures were measured using high performance ion chromatography (IC) (Dionex ICS5000 dual channel, Hemel Hempstead, United Kingdom) as described previously (Nixon et al., 2018). Mean changes in concentration of anions in MPN tests were assessed for significance using a Student *t* test (2-tailed, type 2, critical value of 0.05).

### DNA Extraction and 16S rRNA Gene Amplification and Sequencing

The standard protocol describing 16S rRNA gene sequencing carried out on samples shortly after sample collection is precisely detailed in Borton et al. (2017).

At the time of inoculation of the MPN series, 1 ml samples of inocula were collected and frozen at −20°C. Further 1 ml samples were collected from positive end points at the conclusion of MPN experiments and stored at −20°C. DNA was extracted from 0.2 ml aliquots of these samples using the DNeasy PowerLyzer^TM^ PowerSoil Kit (Qiagen, Manchester, United Kingdom). Positive (Escherichia coli) and negative (ultrapure water) controls were also run. Sequencing of PCR amplicons of 16S rRNA was conducted with the Illumina^®^ MiSeq^TM^ platform (Illumina, San Diego, CA, United States) targeting the V4 hyper variable region (forward primer, 515F, 5′-GTGYCAGCMGCCGCGGTAA-3′; reverse primer, 806R, 5′-GGACTACHVGGGTWTCTAAT-3′) for two 250-bp paired-end sequencing (Caporaso et al., 2011, 2012). PCR amplification was performed using Roche FastStart^TM^ High Fidelity PCR System (Roche Diagnostics Ltd., Burgess Hill, United Kingdom) in 50 μl reactions under the following conditions: initial denaturation at 95°C for 2 min, followed by 36 cycles of 95°C for 30 s, 55°C for 30 s, 72°C for 1 min, and a final extension step of 5 min at 72°C. The PCR products were purified and normalized to ∼20 ng each using the SequalPrep Normalization Kit (Fisher Scientific, Loughborough, United Kingdom). The PCR amplicons from all samples were pooled in equimolar ratios. The run was performed using a 4 pM sample library spiked with 4 pM PhiX to a final concentration of 10% in accordance with Kozich et al., 2013 (Kozich et al., 2013). Raw sequences were divided into samples by barcodes (up to one mismatch was permitted) using a sequencing pipeline as follows. Quality control and trimming was performed using Cutadapt (Compeau et al., 2013), FastQC (Andrews, 2010), and Sickle (Joshi and Fass, 2011). MiSeq^TM^ error correction was performed using SPAdes (Nurk et al., 2013). Forward and reverse reads were incorporated into full-length sequences with Pandaseq (Masella et al., 2012). Chimeras were removed using ChimeraSlayer (Haas et al., 2011), and OTU’s were generated with UPARSE (Edgar, 2013). OTUs were classified by Usearch (Edgar, 2010) at the 97% similarity level, and singletons were removed. Rarefaction analysis was conducted using the original detected OTUs in Qiime (Caporaso et al., 2010). Taxonomic assignment was performed by the RDP naïve bayesian classifier version 2.2 (Wang et al., 2007), used in combination with the Silva SSU 132 ribosomal RNA gene database (Quast et al., 2013). The OTU tables were rarefied to the sample containing the lowest number of sequences, all samples having less than 5,000 sequences were removed from analyses prior to the rarefaction step. The step size used was 2000 and 10 iterations were performed at each step. Both sequencing reaction mix controls and extraction controls were sequenced alongside the samples and any shared OTU’s found to be present in these were manually deleted from the Sample OTU table prior to final analysis.

### Accession Numbers

Raw sequencing data obtained in this study are available at the NCBI Sequencing Read Archive^1^ under the accession numbers PRJNA578032, SAMN13315659, SAMN13315660, SAMN13315661, and SAMN13315662.

## Results

### Most Probable Number Counts

To quantify the numbers of persistent culturable sulfide-producing bacteria in produced waters from the Marcellus shale gas well studied, a series of MPN enrichments were set up targeting sulfate-reducing and TRB (alongside FB) using samples that had been collected 29, 79, 142, and 303 days after hydraulic fracturing, and then stored for 534–808 days at room temperature as shown in Table 1 prior to cultivation. The term “persistent” is used here to describe cells that survive storage conditions and can be cultured under the conditions provided. The formation of black precipitate (iron sulfide) in thiosulfate and sulfate-reducing media was scored as positive and indicative of sulfide production. An increase in turbidity was scored as positive growth in the FB medium. All MPN vials were incubated at 40°C for 112 days prior to scoring.

**TABLE 1:** Most probable number (MPN) counts (cells/ml) after 112 days incubation and the average cell counts from time of sample collection.

	**Days after hydraulic fracturing/storage time**			
	**Day 29/808 days**	**Day 79/758 days**	**Day 142/695 days**	**Day 303/534 days**
Fermentative bacteria	1.1 × 10^6^	1.1 × 10^6^	9.3 × 10^4^	9.3 × 10^4^
Sulfate-reducing bacteria	0	0	0	0
Thiosulfate-reducing bacteria (no yeast extract)	3.6 × 10^0^	9.2 × 10^0^	2.1 × 10^1^	9.2 × 10^0^
Thiosulfate-reducing bacteria (added yeast extract)^a^	9.2 × 10^1^	1.1 × 10^6^	4.2 × 10^3^	9.3 × 10^4^

*^*a*^ Samples described in this row were each stored for an additional 291 days prior to use as inoculum.*

Most probable number counts (Table 1) showed that across all the samples analyzed, FB were more numerous than dissimilatory sulfate- or TRB. In initial MPN tests targeting dissimilatory TRB, using a fully defined selective medium, only low numbers of TRB were cultured in samples (following storage for 534–808 days), with a maximum MPN count of just 21 cells/ml when inoculated with the sample from day 142 post hydraulic fracturing. After an additional 291 days storage, the inoculum was used to repeat the TRB l MPNs with the addition of yeast extract, and MPN counts increased considerably, peaking at 1.1 × 10^6^ cells/ml, despite their prolonged storage. Indeed, MPNs conducted in TRB medium amended with yeast extract were up to several orders of magnitude greater than those conducted in the absence of yeast extract, suggesting that yeast extract has a role in stimulating the growth of TRB.

While the estimated number of persistent FB was initially high (approximately 1.1 × 10^6^) in MPNs inoculated with produced waters collected 29 and 79 days after hydraulic fracturing (and stored for 808 and 758 days respectively), MPN counts were approximately two orders of magnitude lower in the MPNs inoculated with later produced waters (collected 142 and 303 days after hydraulic fracturing and stored for 695 and 534 days, respectively). The MPN counts for TRB in tests with added yeast extract, inoculated with the produced waters collected 79 and 303 days after hydraulic fracturing (and stored for 1049 and 825 days, respectively), approached the MPN counts obtained from FB. The number of persistent TRB estimated in the presence of yeast extract increased from 9.2 × 10^1^ to 1.1 × 10^6^ between produced waters from 29 to 79 days after hydraulic fracturing, and MPN counts were maintained in the range of 4.2 × 10^3^ and 9.3 × 10^4^ for the later samples. The equivalent number of thiosulfate-reducing and FB estimated from MPN tests on samples collected 79 and 303 days post hydraulic fracturing, after hundreds of days storage suggests that the majority of FB detected were also capable of thiosulfate reduction. No culturable SRB were detected in the SRB MPNs across any of the four stored produced waters tested (Table 1).

### Ion Chromatography to Support Thiosulfate Reduction in Relevant MPN Counts

After 112 days incubation the initial TRB MPNs (without yeast extract) showed a statistically significant (*p* < 0.05) decrease in the average concentration of lactate, acetate and thiosulfate relative to abiotic controls. Positive MPN end-points from TRB l medium with the addition of yeast extract also showed a statistically significant (*p* < 0.05) decrease in thiosulfate concentrations after the same incubation period. In addition, positive end points from the thiosulfate-reducing MPNs, with the addition of yeast extract, did not show a significant decrease in the concentration of lactate or acetate after a total of 112 days incubation, despite the significant reduction of thiosulfate, suggesting that here thiosulfate-reduction may instead be coupled to the fermentation of compounds present in yeast extract rather than acetate or lactate metabolism.

### Microbial Community Composition Based on 16S rRNA Gene Sequencing

**FIGURE 1:**
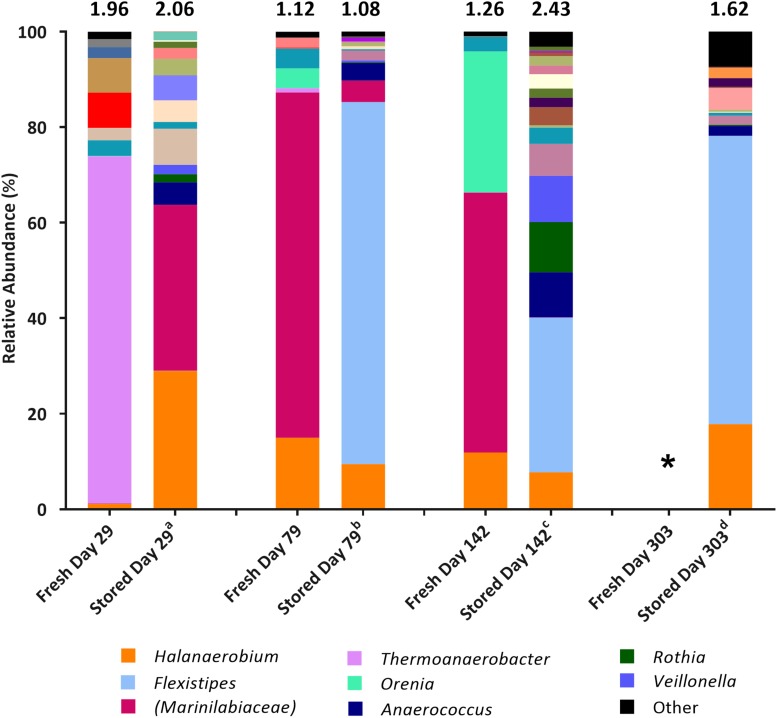
Genus-level microbial community comparison based on 16S rRNA gene sequencing from fresh samples (DNA extracted shortly after collection) compared with stored samples (DNA extracted at the time of initial MPN inoculation). DNA was extracted from fresh samples shortly after sample collection, DNA was extracted from stored samples at the time of initial MPN inoculation. Where genus level could not be resolved the last matched taxonomic level was assigned in brackets. “Other” is comprised of taxa that represent less than 1% relative abundance combined across all samples. The Shannon diversity index calculated from operational taxonomic units is displayed above each bar. ^a^ 808 days storage, ^b^ 758 days storage, ^c^ 695 days in storage, and ^d^ 534 days in storage. * denotes 16S rRNA gene data unavailable. Key taxa are highlighted in legend.

**FIGURE 2:**
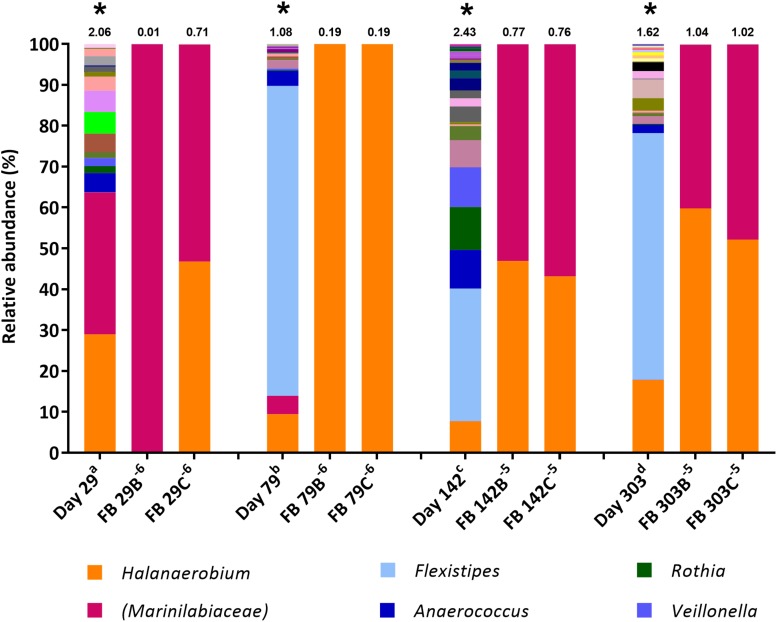
Genus-level microbial community composition of fermentative bacteria (FB) MPN enrichments based on 16S rRNA gene sequencing. Enrichments were conducted at 8% salinity. Where genus level could not be resolved the last matched taxonomic level was assigned in brackets. The Shannon diversity index calculated from operational taxonomic units is displayed above each bar. ^a^ 808 days storage, ^b^ 758 days storage, ^c^ 695 days in storage, and ^d^ 534 days storage. * indicates inocula. Key taxa are highlighted in legend. Enrichments were conducted in triplicate where A, B, C refers to specific replica and superscript power refers to dilution factor of the positive end-points sequenced.

**FIGURE 3:**
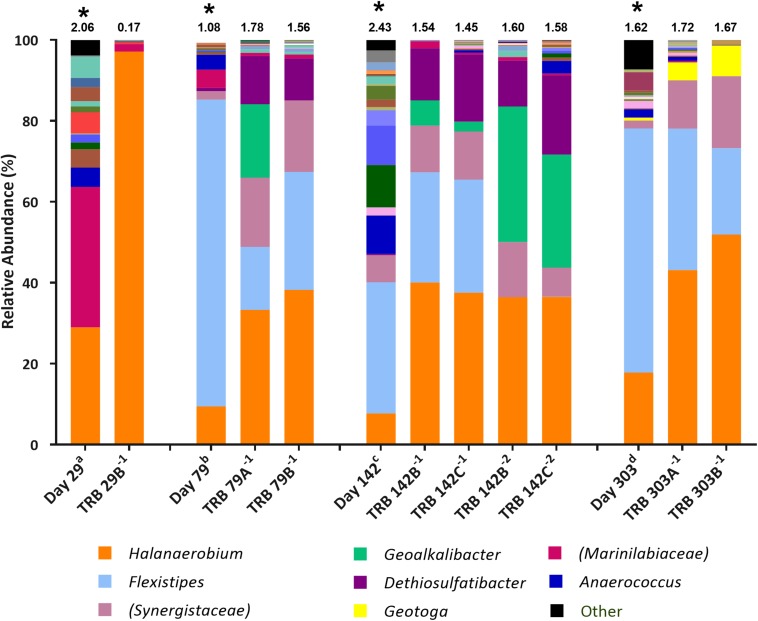
Genus-level microbial community composition of MPN enrichments for TRB based on 16S rRNA gene sequencing. Enrichments were conducted at 10% salinity. Genera that constitute less than 0.1% combined abundance were grouped as “other.” Where genus level could not be resolved the highest resolved taxonomic level is given in brackets. The Shannon diversity index calculated from operational taxonomic units is displayed above each bar. ^a^ 808 days storage, ^b^ 758 days storage, ^c^ 695 days in storage, and ^d^ 534 days storage, * indicates inocula. Key taxa are highlighted in legend. Enrichments were conducted in triplicate where A, B, C refers to specific replica and superscript power refers to dilution factor of the positive end-points sequenced.

**FIGURE 4:**
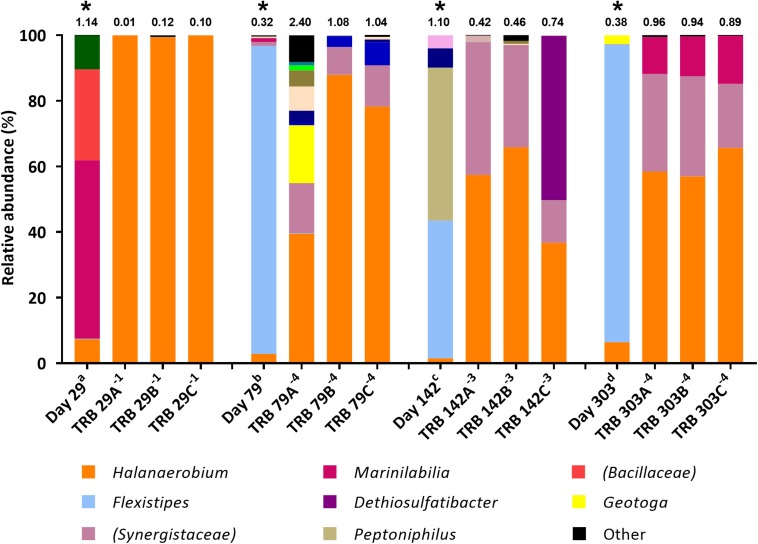
Genus-level microbial community composition of TRB MPN enrichments amended with yeast extract, based on 16S rRNA gene sequencing. Enrichments were conducted at 10% salinity. Where identity at genus level could not be resolved the highest resolved taxonomic level is given in brackets. The Shannon diversity index calculated from operational taxonomic units is displayed above each bar. ^a^ 808 days storage, ^b^ 758 days storage, ^c^ 695 days in storage, and ^d^ 534 days storage, * indicates inocula. Key taxa are highlighted in legend. Enrichments were conducted in triplicate where A, B, C refers to specific replica and superscript power refers to dilution factor of the positive end-points sequenced.

Microbial community composition in enrichment cultures from the positive MPN tubes was assessed using 16S rRNA gene sequencing with the illumina^®^ MiSeq^TM^platform. The aims were to compare how community composition changed between sample collection and their use as inocula in MPN tests reported here (Figure 1), and to assess which taxa became enriched in MPN tests targeting fermentative (Figure 2), sulfate-reducing and thiosulfate-reducing (Figures 3, 4) microorganisms. 16S rRNA gene sequencing was conducted on all positive MPN end-points. As such, data are presented for fermentative and thiosulfate-reducing enrichments only, as no SRB l MPNs were scored as positive. In the figures the Shannon H diversity index is displayed above each bar as a measure of change in diversity between samples.

### Persistent Community Members in Aged Produced Water Samples

Community composition analysis showed few similarities at the time of sample collection, compared to when the stored samples were sequenced at the time of initial MPN inoculation, some 534–808 days after sample collection (Figure 1). Generally, species diversity increased during sample storage, likely a result of previously dominant taxa diminishing in numbers over time so that lower abundance (previously undetected) taxa represented a larger proportion of the remaining community. Sequences closely associated to the prevailing taxa *Halanaerobium* and *Marinilabiaceae* were present at the time of inoculation, but generally decreased in relative abundance over time (comparing community compositions in freshly sampled waters, and those of the stored samples used as inocula) with the exception of the Day 29 sample. Taxa not detected at the time of collection, or that were present only at low relative abundance (such as *Anaerococcus* and *Flexistipes*), represented a higher proportion of the microbial population present at time of inoculation (Figure 1). SRB affiliated with the genera *Desulfotomaculum* and *Desulfovibrio*, identified at the time of sample collection (combined relative abundance of <3.43% across all samples), were not detected in the inoculum.

### Community Composition of Positive Fermentative MPNs

The FB medium (Spring et al., 2015) used in the MPN cultures for fermentative microorganisms is a non-restrictive sugar-containing medium containing both the fermentable carbon substrate glucose and yeast extract as a source of additional nutrients. MPNs in FB medium exhibited rapid and extensive growth (observed as turbidity) compared with MPNs initially set up using the thiosulfate-reducing and sulfate-reducing media. Figure compares the microbial community compositions of produced water inocula to the highest dilution FB MPN enrichments that were scored positive.

The diversity of 16S rRNA genes was lower in positive enrichments, compared to the inocula, with a Shannon *H* index as low as 0.01 in one enrichment (Figure 2), dominated almost entirely by OTUs belonging to the family *Marinilabiaceae* (99.91% relative abundance). Organisms most closely affiliated with *Marinilabiaceae* were detected across the inocula at relative abundances ranging from 0.02% in the stored day 303 sample to 34.7% in the stored day 29 sample (stored for 534 and 808 days, respectively, before use as MPN inocula). Although stored produced water collected 142 and 303 days post hydraulic fracturing contained less than 1% *Marinilabiaceae*, this increased to 53–56% and 40–47%, respectively, when transferred to FB medium. Organisms most closely related with the family *Marinilabiaceae* or genus *Halanaerobium* appear to be similarly dominant, with a combined abundance of over 99.8% in all enrichments. MPN enrichments for FB inoculated with the stored produced water collected 79 days post hydraulic fracturing were dominated by members of the genus *Halanaerobium*, which comprised over 99.9% relative abundance.

### Community Composition of Positive Thiosulfate-Reducing MPNs

*Halanaerobium* species were found to increase in relative abundance across thiosulfate-reducing enrichments compared with inocula (Figure 3). The highest enrichment of *Halanaerobium* species was observed in MPNs inoculated with the day 29 produced water (30.0% relative abundance), with a final relative abundance of 97.2% in the MPN enrichment, thus highlighting *Halanaerobium* as a key player in sulfide production. Compared to the inocula, the relative abundance of *Dethiosulfatibacter* species increased ten-fold in all enrichments. Similarly *Geoalkalibacter* species, not detected in the inocula, were observed in positive enrichments inoculated with day 79 and day 142 produced waters. Organisms most closely affiliated with *Geotoga* species increased in relative abundance in TRB MPNs inoculated with the day 303 sample from less than 0.1% to 7.63%. *Flexistipes* species declined in relative abundance in TRB MPNs, but still remained the second most abundant genus after *Halanaerobium* at a dilution of 10^–1^ in all enrichments. *Marinilabiaceae* was detected in all starting inocula but their relative abundance declined across all enrichments to below 2%.

Subsequent TRB MPN enrichments amended with yeast extract were inoculated 9 months after the initial TRB MPNs. As such, community composition of these older inocula were assessed with 16S rRNA gene sequencing. Compared with the inocula used in initial TRB MPNs (Figure 3), Shannon diversity *H* index had decreased by the time of their use in yeast extract-amended TRB MPNs (Figure 4). Generally the relative abundance of *Halanaerobium* species in the MPN inocula had decreased over time while the relative abundance of *Flexistipes* species had increased over time.

Addition of yeast extract to TRB medium led to comparable community composition in positive enrichments (Figure 4) to that of FB enrichments (Figure 2). In all yeast extract-amended TRB positive enrichments, the relative abundance of *Halanaerobium* species increased, becoming the dominant lineage in most cases (Figure 4). Similarly, members of the family *Synergistaceae* increased in relative abundance from below 2.0% in the inocula to between 8.4% and 40.5% relative abundance in positive thiosulfate-reducing MPNs inoculated with day 142 and 303 produced waters, exhibiting up to a 6-fold increase in relative abundance (Figure 4).

In contrast to TRB positive MPNs without added yeast extract, the relative abundance of organisms most closely affiliated with *Flexistipes* dropped to below 0.2%. *Dethiosulfatibacter* was undetected in inocula yet became the dominant taxon in one positive enrichment inoculated with day 142 sample (50.0%).

## Discussion

Hydraulic fracturing as a means of recovering natural gas from shale is known to create new microbial ecosystems in the deep subsurface (Daly et al., 2016). Some microbial processes in fractured shales are known to negatively impact on natural gas extraction, for instance through hydrocarbon souring and microbial-induced corrosion (Johnson et al., 2008). Here, we assessed the presence of fermentative and sulfidogenic bacteria in shale gas production fluids with a focus on persistent species surviving several hundred days in storage. Using a targeted MPN enrichment approach coupled to DNA-based microbial composition analysis, we demonstrated that dominant halotolerant taxa in fractured shales are persistent and have the potential to sour gas, contribute to corrosion and proliferate in the presence of fermentative substrates, potentially reducing permeability and thus natural gas recovery.

Microbial community composition of the production fluids shows a marked shift following storage, with communities changing between sample collection and initial testing (Figure 1), and further changes occurring between initial MPNs (Figures 2, 3) and follow up MPNs (Figure 4). After the long periods since their collection, produced water samples used in MPN enrichments still contained relatively high abundances of prominent taxa previously reported in fractured shale ecosystems, including species of *Halanaerobium* and *Marinilabiliaceae* (Wuchter et al., 2013; Fichter et al., 2017). Persistent microorganisms identified here were able to withstand nutrient-limiting storage conditions and grow in targeted MPN enrichments.

Fermentative and thiosulfate-reducing microorganisms were observed in MPN enrichments inoculated with all production fluids. In contrast, although 16S rRNA gene sequencing detected sequences affiliated with sulfate-reducing microorganisms at the time of sample collection, none were detected through MPN culturing (Table 1), in line with previous studies (Struchtemeyer and Elshahed, 2012). Fermentative enrichments were dominated by members of the *Halanaerobium* genus and *Marinilabiliaceae* family (Figure 1). This is unsurprising given that FB medium was the only selective growth medium containing glucose as members of both taxa can utilize glucose (Denger et al., 2002; Liang et al., 2016). Initial thiosulfate-reducing MPN enrichments led to increased abundances of taxa affiliated with *Geoalkalibacter*, *Geotoga*, *Dethiosulfatibacter*, and *Synergistaceae* (Figure 3), although subsequent enrichments amended with yeast extract were dominated by – *Halanaerobium* (Figure 4). Collectively, these findings demonstrate that a diverse array of fermentative and sulfidogenic microorganisms remain viable in production fluids for long periods of time. Although not all taxa putatively identified through 16S rRNA gene sequencing were culturable within the selective media used, many were amenable to laboratory culturing and quantification. The authors acknowledge that the metabolic activity witnessed under laboratory conditions may not reflect metabolic activity *in situ*.

### Persistent Sulfidogenic Taxa

No SRB were observed in MPN tests (Table 1), despite their detection in produced waters shortly after collection. Sequences affiliated with SRB have been detected in drilling muds and produced water impoundments (Struchtemeyer et al., 2011; Murali Mohan et al., 2013b) and transcriptomic evidence suggests that the sulfate reduction pathway is active in produced waters (Vikram et al., 2016). The lack of viable SRB cultured from the production fluids following storage, suggests they are not persistent taxa and therefore pose limited threat long-term when considering the recycling of produced waters after extended holding periods. However, we acknowledge that the culturing conditions used may not have been conducive to the growth of all SRB.

Although sulfate-reducing MPNs were scored negative, a number of sulfidogenic taxa were observed in thiosulfate-reducing enrichments, including representatives of the genera *Halanaerobium*, *Dethiosulfatibacter, Geoalkalibacter*, and *Geotoga* (Figures 3, 4). It is well known that *Halanaerobium* species are able to colonize hydraulically fractured shale ecosystems, regardless of geography (Mouser et al., 2016; Lipus et al., 2017) and are ubiquitous across shale gas wells in the United States (Struchtemeyer and Elshahed, 2012; Murali Mohan et al., 2013a; Strong et al., 2013; Cluff et al., 2014; Akob et al., 2015; Daly et al., 2016; Mouser et al., 2016; Booker et al., 2017; Lipus et al., 2017; Borton et al., 2018). The moderate halophile *Halanaerobium congolense* has also been shown to reduce both thiosulfate and elemental sulfur to sulfide, but lacks the ability to reduce sulfate to sulfide (Ravot et al., 2005). Metabolic pathway analyses have revealed that *Halanaerobium* species have the potential for thiosulfate reduction, acid production and biofilm formation (Lipus et al., 2017), potentially making this a highly problematic microorganism during shale gas extraction. Indeed this ability has led to an emerging interest in the role that *Halanaerobium* species may have in the biogenesis of sulfide and corrosive organic acids, such as acetate (Booker et al., 2017, 2019). It is therefore unsurprising that *Halanaerobium* spp. were detected across all produced water samples studied here, and showed increased relative abundance across all targeted enrichments, even after prolonged periods in storage.

*Dethiosulfatibacter*, another known thiosulfate-reducing bacterium (Takii et al., 2007; Qian et al., 2019), was also enriched in positive TRB MPNs, with and without yeast extract, despite low abundance in the inocula (Figures 3, 4). Members of this genus were also detected in production fluids from a 4.1 km shale gas well in the Sichuan Basin, China, and relative abundance was found to increase between produced water production and storage (Zhang et al., 2017). Together these findings indicate *Dethiosulfatibacter* may contribute to corrosion and souring during shale gas extraction.

Also enriched in thiosulfate-reducing enrichments was *Geoalkalibacter*. Although not detected in produced water samples, its increased abundance in enrichments from day 79 to day 142 samples suggest that it is capable of thiosulfate reduction. *Geoalkalibacter* was not detected in any enrichments that used yeast extract containing media, despite reports that yeast extract is required as a source of growth factors (Zavarzina et al., 2006), perhaps due to being outcompeted by dominant *Halanaerobium* strains (Figure 4). Isolates of this genus have not been shown to mediate thiosulfate reduction, although *G. ferrihydriticaus* and *G. subterraneus* can produce sulfide from the reduction of elemental sulfur (Zavarzina et al., 2006; Greene et al., 2009). *Geoalkalibacter* species have been identified previously in produced waters collected from shale gas wells in the Antrim shale formation (Wuchter et al., 2013). When taken together, these findings suggest members of this genus may in fact be capable of thiosulfate reduction, and could play a role in souring and microbially induced corrosion during shale gas extraction.

Members of the genus *Geotoga* are also capable of reducing elemental sulfur to form hydrogen sulfide (Davey et al., 1993), but to the best of our knowledge is not known to be capable of thiosulfate reduction. *Geotoga* species were detected at low levels (<1%) in the initial day 79 and 303 produced water samples, but showed a ten-fold increase in relative abundance when cultured in thiosulfate-reducing media (Figure 3). *Geotoga* species were observed in similar sulfidogenic enrichments using produced waters from the Marcellus shale, although the added electron acceptor in that study was sulfate in the form of barite (Wuchter et al., 2013). Laboratory reactor experiments established with Utica produced waters and amended with glycine betaine also gave rise to an enrichment in *Geotoga* (Borton et al., 2018). Proteomic evidence for carbon-based metabolism in *Geotoga* in these experiments highlighted its importance in persistent shale metabolic networks (Borton et al., 2018). Our findings also demonstrate it is a persistent member of fractured shale communities, and may also contribute to biogenic sulfide production.

Evidently, many sulfide producing taxa have the potential to remain persistant and metabolically active for many years post fracturing, as illustrated through the community composition analysis of positive thiosulfate-reducing enrichments (Figures 3, 4). Although SRB have been identified previously in production fluids through next generation sequencing (Davis et al., 2012; Struchtemeyer and Elshahed, 2012; Liang et al., 2016) no SRB were shown to be viable in these experiments, in agreement with Struchtemeyer and Elshahed (2012). Multiple *Halanaerobium* species have been isolated from shale environments and exhibit thiosulfate-reducing capabilities (Ravot et al., 1997, 2005; Fichter et al., 2012; Liang et al., 2014, 2016; Booker et al., 2017). It appears more likely that thiosulfate-reduction is the more prominent sulfidogenic pathway in fractured shale communities, as evidenced by the widespread presence of the thiosulfate-reducing bacterium *Halanaerobium* (Davis et al., 2012; Struchtemeyer and Elshahed, 2012; Murali Mohan et al., 2013a; Strong et al., 2013; Cluff et al., 2014; Akob et al., 2015; Daly et al., 2016; Mouser et al., 2016; Vikram et al., 2016; Booker et al., 2017; Lipus et al., 2017; Borton et al., 2018; Evans et al., 2019).

Biogenic sulfide production in conventional oil reservoirs has long been attributed to the activity of SRB, and targeted culture-based MPN enrichments for these organisms have been effective in diagnosing a potential biogenic souring problem. Molecular analyses targeting genes in the sulfate reduction pathway have also been described (Muyzer and Stams, 2008). However, in line with our own findings, these approaches will not detect the diverse array of taxa in fractured shale communities capable of sulfide production via thiosulfate reduction. The long-term persistence of sulfidogenic lineages demonstrated in this study further highlights the need for effective diagnostic tools and control strategies targeted to thiosulfate-reducing microorganisms.

### Influence of Yeast Extract

Yeast extract was added as a source of trace nutrients to the second round of thiosulfate-reducing MPNs. However, given the strong enrichment for *Halanaerobium* in yeast extract-amended thiosulfate-reducing enrichments, the yeast extract may have served directly as a growth substrate for *Halanaerobium* (Figure 4). MPN counts were several orders of magnitude lower in the initial TRB medium compared with fermentative MPNs cultured in FB medium and thiosulfate-reducing MPNs supplemented with yeast extract (Table 1), confirming that yeast extract enhances growth in these cultures.

Most probable number enrichments conducted in TRB medium with the addition of yeast extract visually formed iron sulfide within days of inoculation, as opposed to months in thiosulfate-reducing MPNs devoid of yeast extract. In contrast to initial thiosulfate-reducing MPN enrichments, no significant decrease in acetate or lactate was observed in yeast-amended thiosulfate-reducing enrichments, suggesting components of yeast extract were serving as alternative electron donors coupled to thiosulfate reduction. Indeed, prior work suggests that *Halanaerobium* uses thiosulfate as a sink for reducing equivalents during fermentation reactions, although this process was not coupled directly to growth (Booker et al., 2017). Yeast extract has also been linked previously to increased sulfide production via thiosulfate reduction, and it has been suggested that yeast extract may itself serve as an electron donor (Liang et al., 2014). One plausible explanation for this is that amino acids within yeast extract could be being fermented by *Halanaerobium*. Amino acids such as lysine and alanine have been suggested as fermentable substrates for *Halanaerobium* based on metagenomic data and these amino acids are likely present within yeast extract (Daly et al., 2016; Borton et al., 2018). Yeast extract may also provide additional growth factors or trace nutrients that are otherwise in limited supply and further accelerate growth rate.

### Other Noteworthy Taxa

In addition to the taxa discussed above, which are likely to catalyze thiosulfate reduction, other microbes that require further discussion include members of the genus *Flexistipes* and family *Marinilabiaceae.* Following storage, members of the genus *Flexistipes* increased in relative abundance, becoming the most abundant taxa at 79, 142, and 303 days post hydraulic fracturing, prior to inoculation in the MPN series (Figure 1). The genus *Flexistipes* has been previously identified in deep marine environments and are strict anaerobes with a requirement for NaCl and a short generation time of just 8.5 h under optimum conditions, so it is unsurprising that they are able to dominate the stored samples (Fiala et al., 1990; Lapidus et al., 2011). However, their relative abundance decreased dramatically when transferred into MPN cultures; for example, produced waters collected 79 and 303 days after hydraulic fracturing and stored for 1049 and 825 days, respectively, prior to use as MPN inocula consisted of over 90% *Flexistipes*, this dropped to below, 0.2% when transferred into thiosulfate-reducing medium containing yeast extract (Figure 4). Based on these observations it is unlikely that *Flexistipes* play a direct role in sulfide production as they were out compete by *Halanaerobium* despite their initial high abundance. However, this drop in relative abundance may also be attributed to unsuitable growth media. FB enrichments were dominated by the members of the family *Marinilabiaceae* and/or genus *Halanaerobium*, able to proliferate despite the high salinity (8% NaCl), moderate temperature (40°C) and the availability of electron donors/acceptors within the FB medium. However, when transferred to thiosulfate-reducing MPN cultures, the relative abundance of *Marinilabiaceae* declined dramiatically across all enrichments to below 2%, suggesting that they are unlikley to play a role in sulfide production, this conclusion is supported by studies showing strains of *Marinilabiaceae* being incapable of sulfide production via thiosulfate reduction (Yang et al., 2014). Such taxa could still have a role in biofouling through fermentation of organic substrates or fracture clogging, for example.

## Conclusion

In summary, this work has shown that many metabolically active sulfide-producing taxa can persist for long periods of time post fracturing, including *Halanaerobium* and *Dethiosulfatibacter* species. Such microorganisms may produce hydrogen sulfide, a chemical which is hazardous to human health (Guidotti, 2010) and can cause souring reactions resulting in sour gas and the corrosion of steel infastructure (Eden et al., 1993; Fichter et al., 2012). Although classical SRB were not abundent in the aged produced waters, a key finding in this study was the identification of the TRB *Halanaerobium*, which was highlighted as a potentially problematic and persistent microorganism within aged produced water communities. This persistence could have significant implications in regard to both sulfide production and biofouling when considering the storage or recycling of produced waters, and needs to be studied in more depth using samples stored appropriately on site. Although produced water is treated prior to reuse with biocides and/or aeration these problematic microorganisms could still go on to seed new wells and some studies suggest that this could lead to a rise in biocide resistance (Russell, 1995; Struchtemeyer and Elshahed, 2012; Murali Mohan et al., 2013b) Clearly new detection and control methods must be installed to combat the problems posed by such persistent and problematic microorganisms. Such studies are now ongoing using cultures from a range of hydraulically fractured shale systems, facilitated by the relative ease of laboratory cultivation of appropriate model organisms such as *Halanaerobium* using selective media, as demonstrated in this study.

## Data Availability Statement

The datasets generated for this study can be found in NCBI SRA accession PRJNA578032 refers to the microcosm experiments and SAMN13315659, SAMN13315660, SAMN13315661, and SAMN13315662 refer to samples “Fresh Day” 29, 79, 142, and 303, respectively.

## Author Contributions

LC, SN, and JL contributed to conception and design of the experiments. RD collected initial samples. MW and KW provided the samples for experiments conducted by LC. DNA was extracted from experiments by LC and 16S rRNA gene sequencing carried out by CB. LC carried out the data analysis, prepared figures and LC and SN wrote the manuscript with contributions and revisions from all authors.

## Conflict of Interest

BE was employed by Rawwater Engineering Company Limited. Authors declare that this study received funding from Rawwater Engineering Company Limited as CASE sponsors for LC’s NERC-funded Ph.D., providing background industry context to the work and comments on the manuscript.

The remaining authors declare that the research was conducted in the absence of any commercial or financial relationships that could be construed as a potential conflict of interest.

## Footnotes

1 https://www.ncbi.nlm.nih.gov/sra/
